# Lipoma of the placenta

**DOI:** 10.1007/s00404-023-07039-z

**Published:** 2023-04-13

**Authors:** Dimitrios Papoutsis, Dimitra Kentrifilli

**Affiliations:** 1 Midwifery Department, School of Health Sciences, University of Western Macedonia, Kozani, Greece; 2 National and Kapodistrian University of Athens, Athens, Greece

## Presentation

**Fig. 1:**
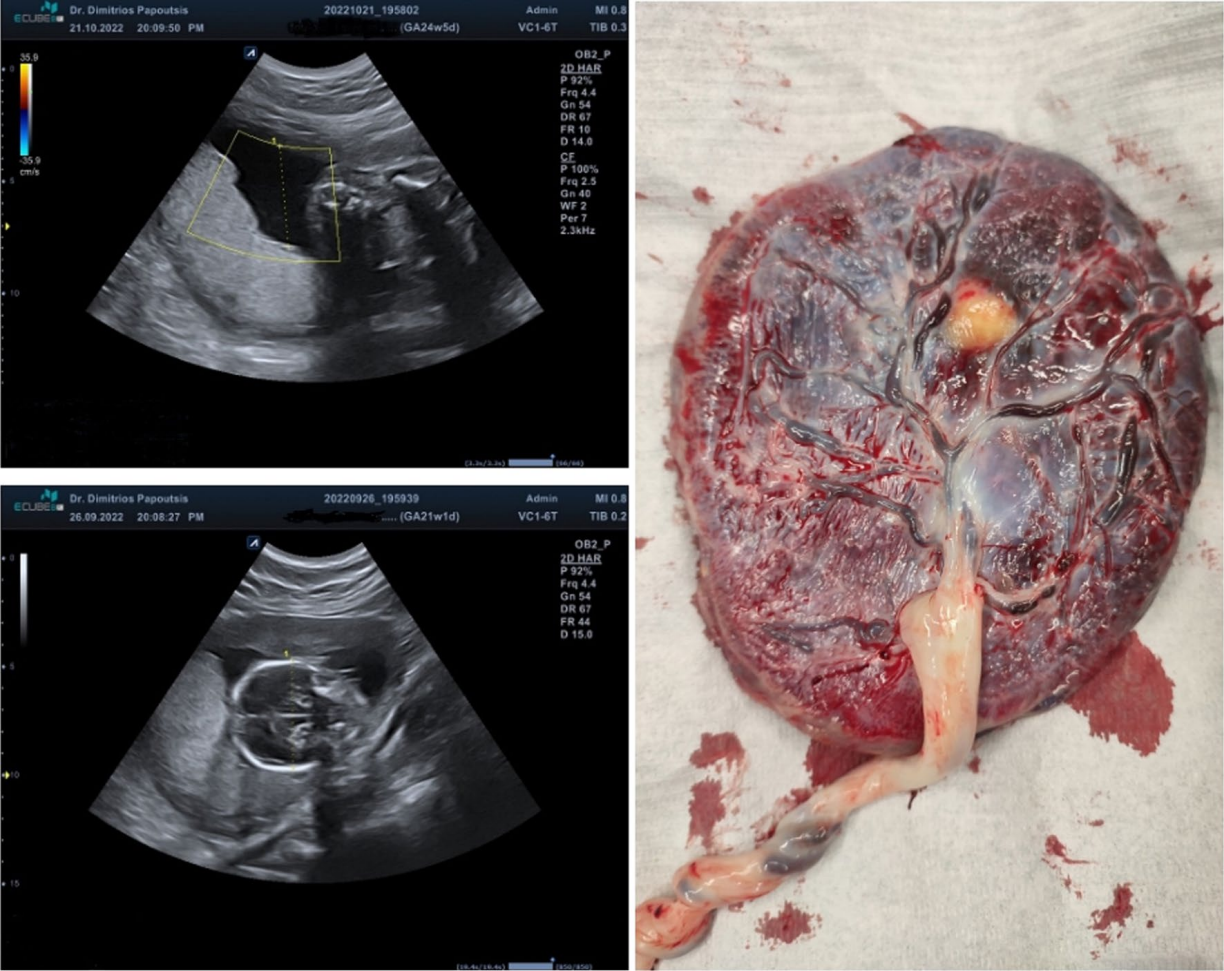
Lower left/upper left: Images of the placenta during routine ultrasound examinations at 21 and 24 weeks of gestation. There was no suspicion of any placental lesion and fetal biometry was within normal range according to gestational age. Right: The placenta in the operating room following delivery of the newborn with a well-circumscribed yellowish nodule measuring 2 × 2 cm consistent with a placental lipoma

A 35-year-old G3P2 was delivered at 35 + 5 weeks of gestation by intrapartum cesarean section due to an abnormal cardiotocogram while in the latent phase of a spontaneous-onset preterm labor. A female neonate was delivered with birthweight of 2400 g (50th centile), Apgar score 8/9 at 1 and 5 min, clear amniotic fluid, and cord blood pH values of 7.18 (arterial) and 7.26 (venous). The neonatal course was uneventful. The patient’s obstetrical history involved two previous cesarean sections and she was opting for a vaginal birth after cesarean section in the current pregnancy. She was diagnosed with antiphospholipid syndrome since her second pregnancy for which she was currently receiving low-molecular weight heparin and low-dose aspirin. In the index pregnancy, she developed gestational diabetes mellitus that was diet controlled. Pre-pregnancy body mass index was 30.8 kg/m^2^ and the weight gain by the time of delivery was 8.5 kg. Fetal growth was uncompromised during the course of gestation. There were no abnormal or suspicious sonographic findings throughout all ultrasound examinations conducted during pregnancy that were indicative of any placental lesion (Fig. 1). Following delivery of the newborn, routine visual and manual inspection of the placenta in the operating room revealed a normal sized unremarkable placenta. However, partially protruding from within the parenchyma there was a well-circumscribed, yellowish, soft-palpable nodule measuring 2 × 2 cm (Fig. 1), that was consistent with a placental lipoma.

## Discussion

There are very few reports in the literature on placental heterotopic tissue, with all of them involving ectopic hepatic or adrenal tissue [1, 2]. Heterotopic adipose tissue in the placenta is extremely rare with only one previous report in the literature [3]. We observed no complications during the neonatal period, which supports the fact that there are no reports on poor outcomes directly related to ectopic tissue in the basal plate of the placenta [4]. In addition, there is no recognized pathophysiologic mechanism that may potentially explain the presence of an intraplacental ectopic mass except for maternal metastatic disease [3]. Since the risk of recurrence in a future pregnancy is currently unknown in the literature and any close proximity of a placental mass near the umbilical cord has been clearly linked to neonatal morbidities and fetal demise [5], we advised this woman to have a targeted and detailed sonographic examination of the placenta in the management of her next pregnancy.

## Data Availability

Data sharing is available upon reasonable request from the corresponding author.
